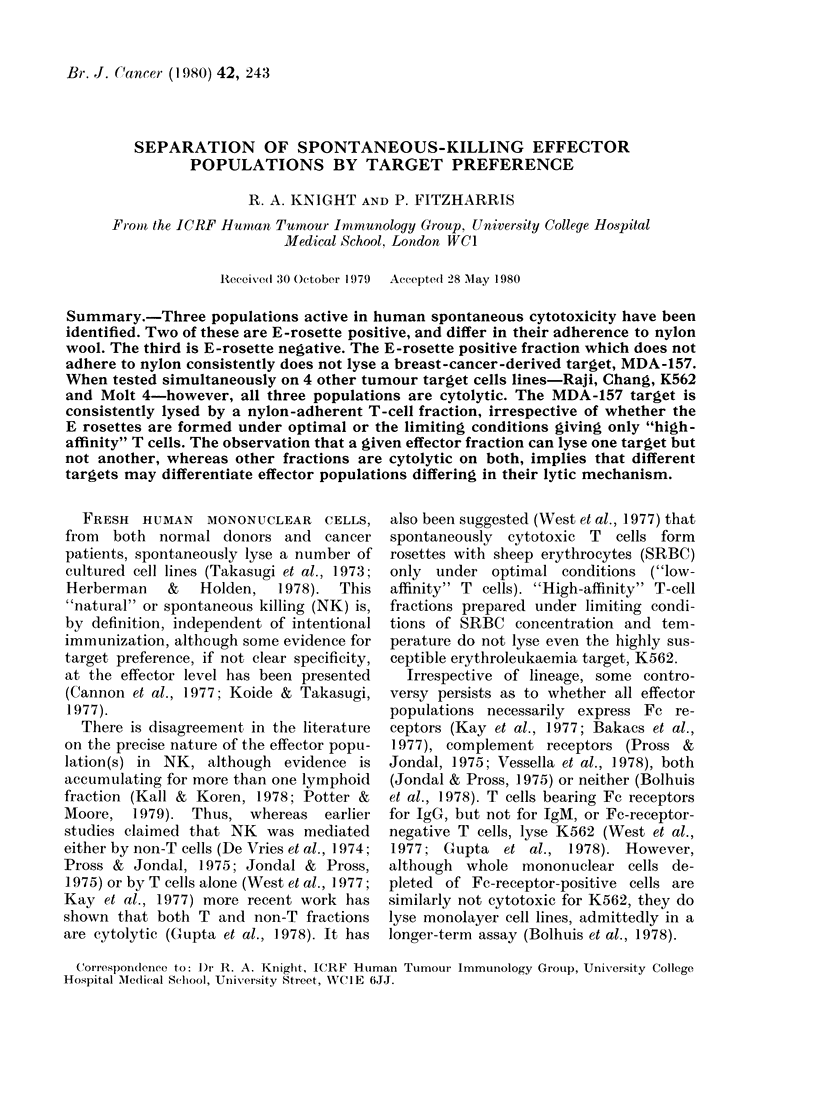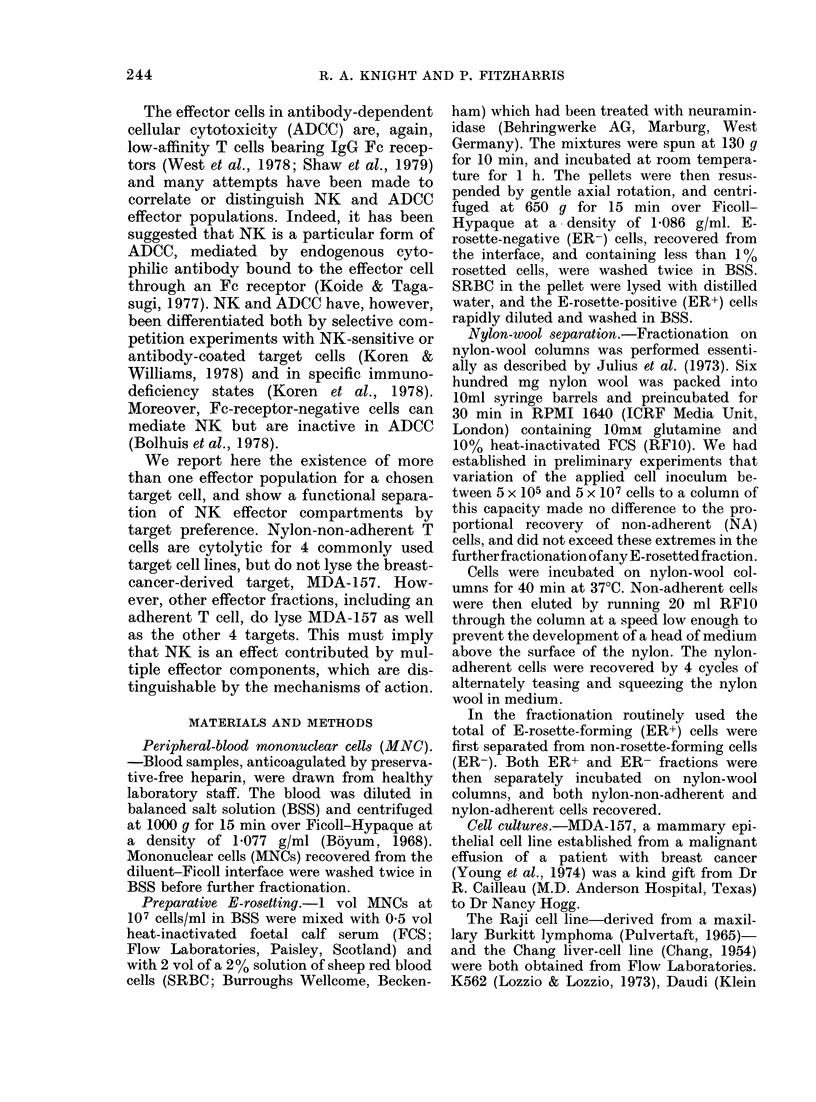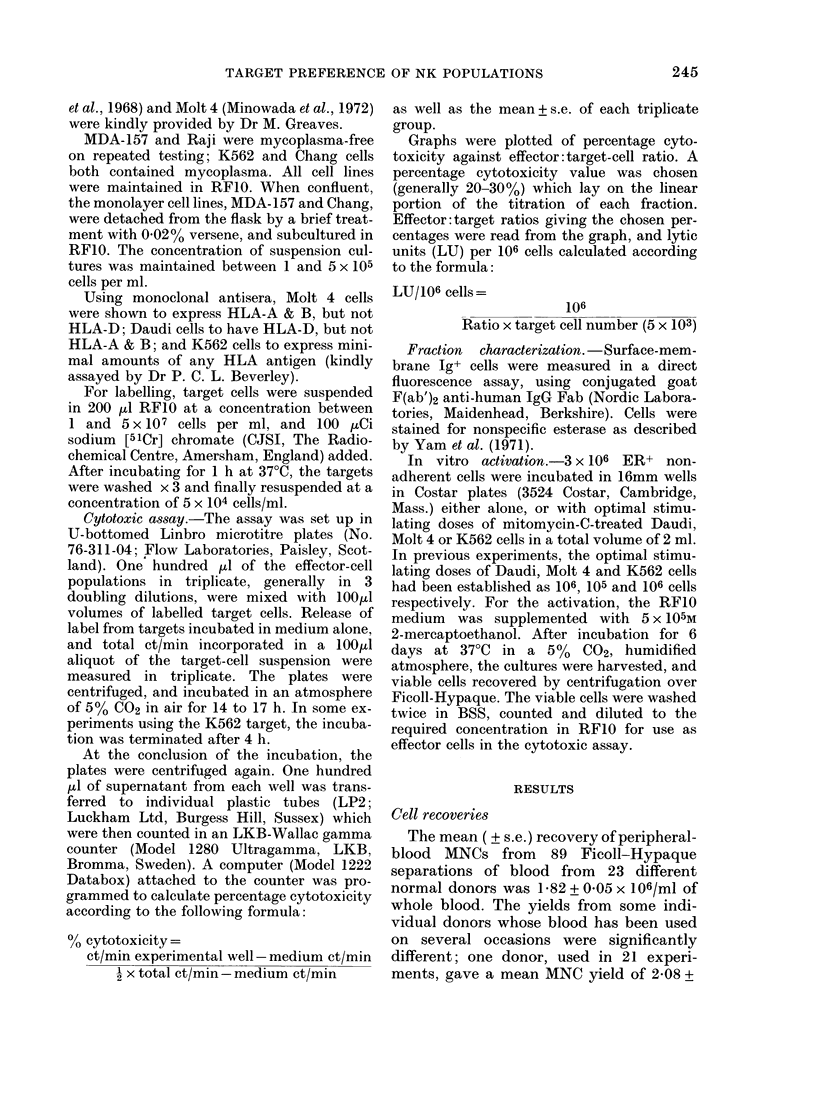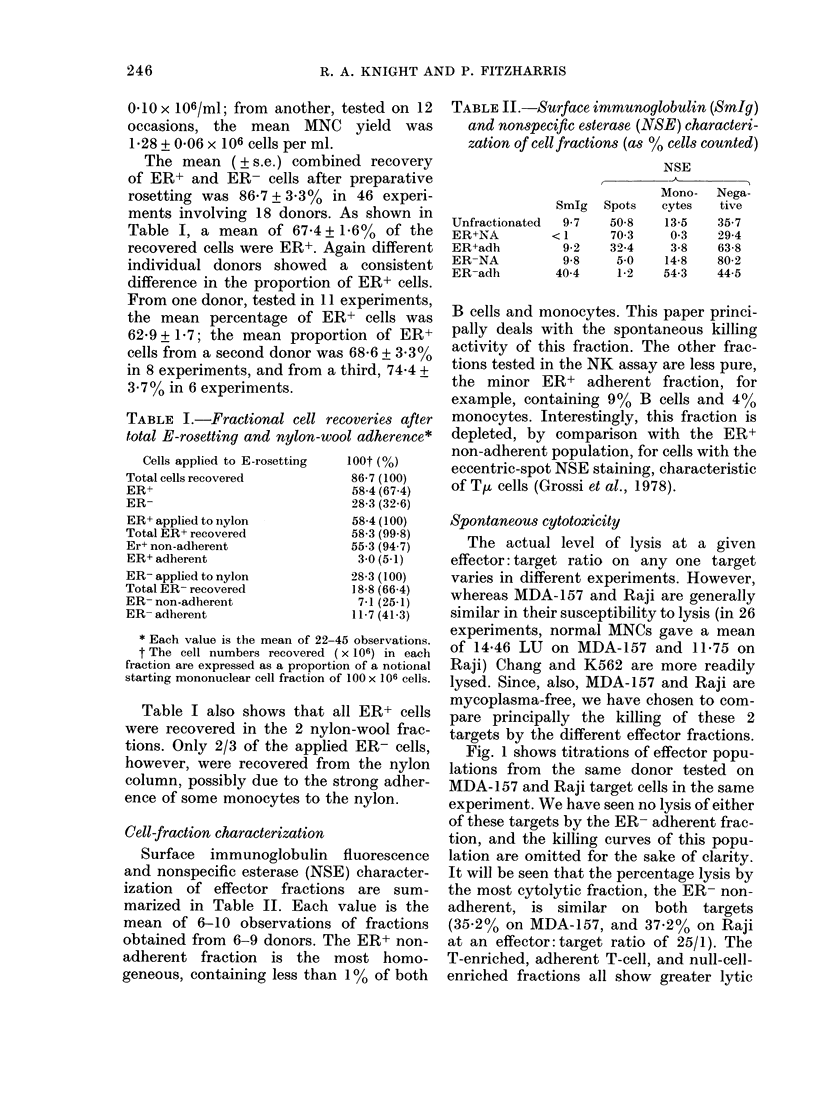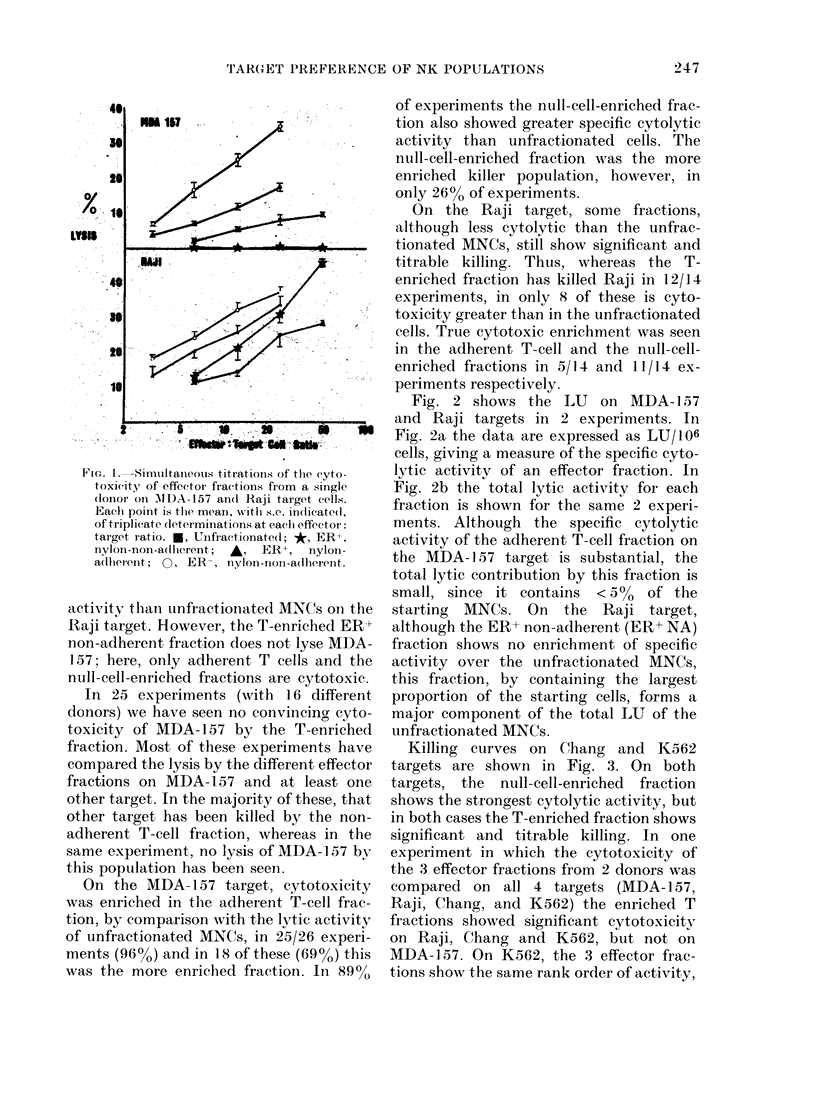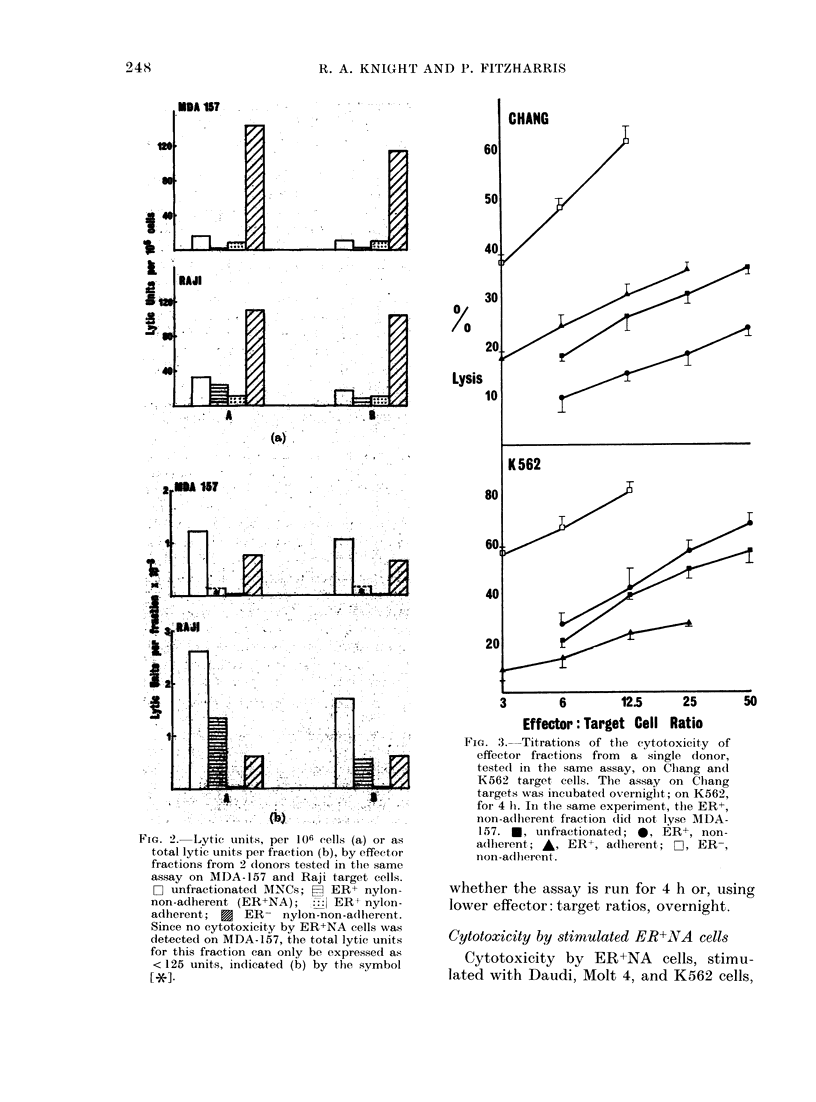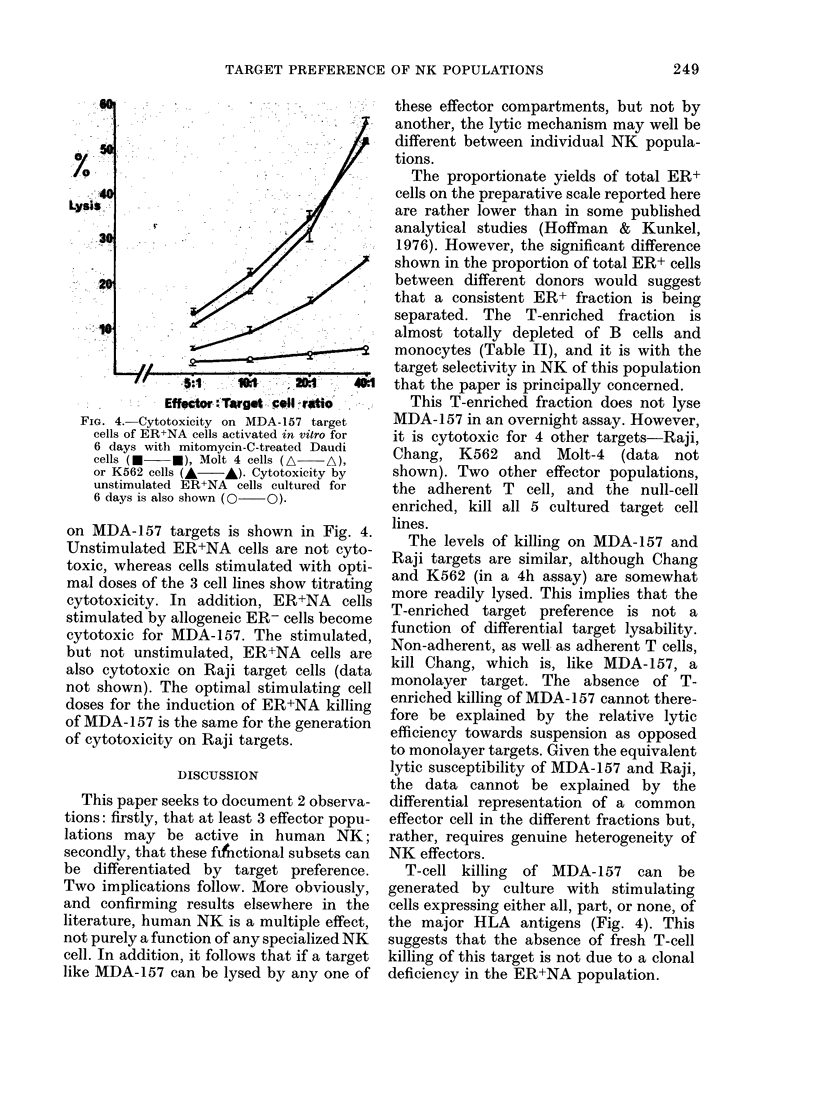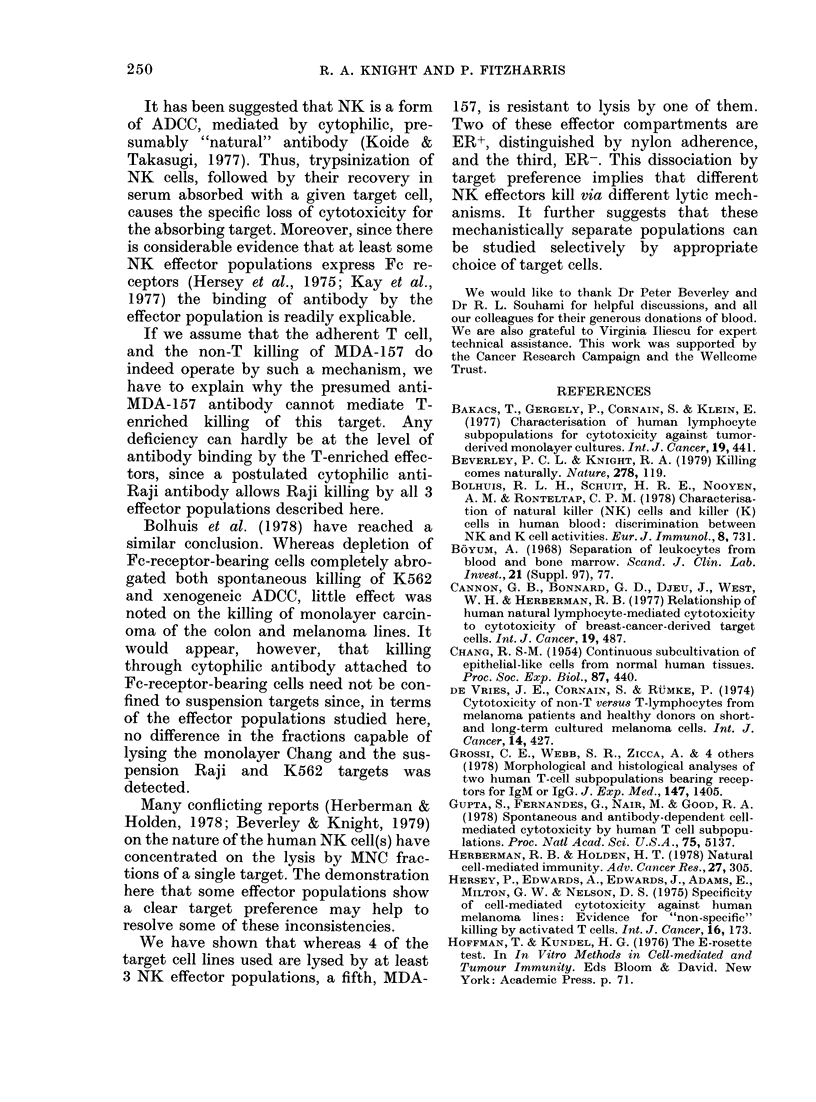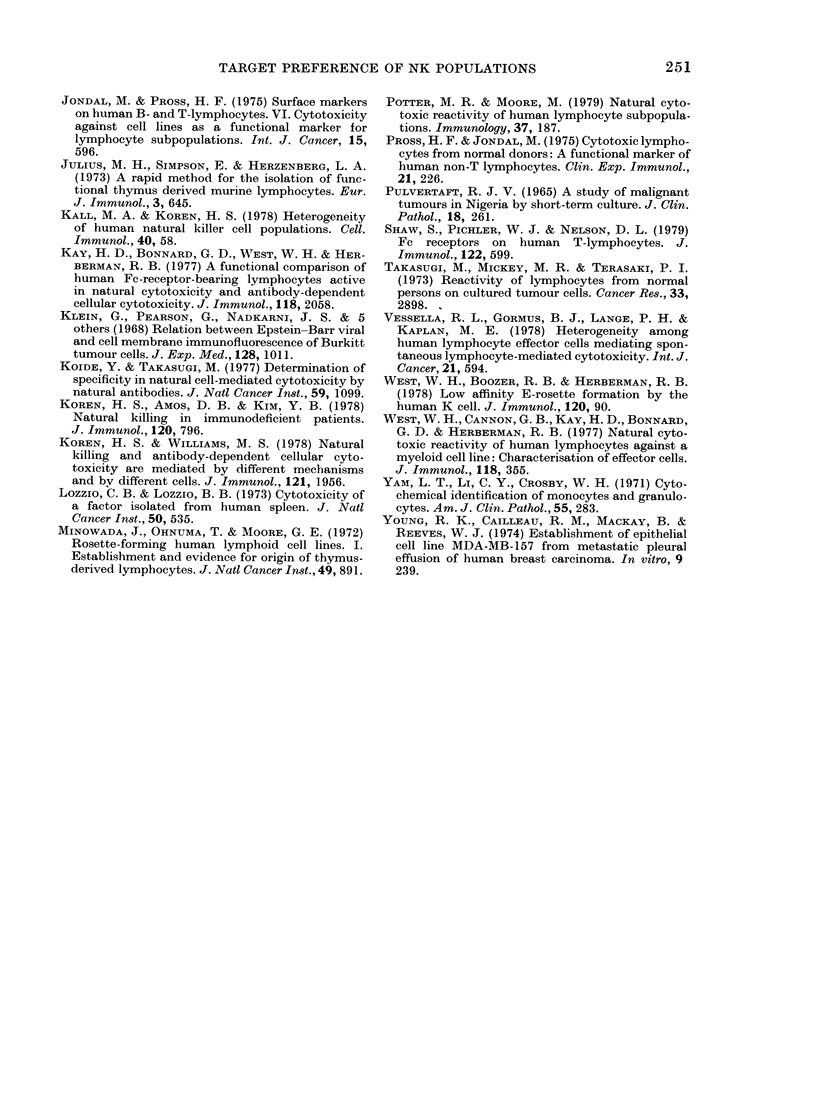# Separation of spontaneous-killing effector populations by target preference.

**DOI:** 10.1038/bjc.1980.223

**Published:** 1980-08

**Authors:** R. A. Knight, P. Fitzharris

## Abstract

Three populations active in human spontaneous cytotoxicity have been identified. Two of these are E-rosette positive, and differ in their adherence to nylon wool. The third is E-rosette negative. The E-rosette positive fraction which does not adhere to nylon consistently does not lyse a breast-cancer-derived target, MDA-157. When tested simultaneously on 4 other tumour target cells lines--Raji, Chang, K562 and Molt 4--however, all three populations are cytolytic. The MDA-157 target is consistently lysed by a nylon-adherent T-cell fraction, irrespective of whether the E rosettes are formed under optimal or the limiting conditions giving only "high-affinity" T cells. The observation that a given effector fraction can lyse one target but not another, whereas other fractions are cytolytic on both, implies that different targets may differentiate effector populations differing in their lytic mechanism.


*Br. J. Cancer* (1980) **42**, 243

### SEPARATION OF SPONTANEOUS-KILLING EFFECTOR POPULATIONS BY TARGET PREFERENCE

R. A. KNIGHT AND P. FITZHARRIS

*From the ICRF Human Tumour Immunology Group, University College Hospital Medical School, London WC1*

Received 30 October 1979 Accepted 28 May 1980

**Summary.—Three populations active in human spontaneous cytotoxicity have been identified. Two of these are E-rosette positive, and differ in their adherence to nylon wool. The third is E-rosette negative. The E-rosette positive fraction which does not adhere to nylon consistently does not lyse a breast-cancer-derived target, MDA-157. When tested simultaneously on 4 other tumour target cells lines—Raji, Chang, K562 and Molt 4—however, all three populations are cytolytic. The MDA-157 target is consistently lysed by a nylon-adherent T-cell fraction, irrespective of whether the E rosettes are formed under optimal or the limiting conditions giving only "high-affinity" T cells. The observation that a given effector fraction can lyse one target but not another, whereas other fractions are cytolytic on both, implies that different targets may differentiate effector populations differing in their lytic mechanism.**

FRESH HUMAN MONONUCLEAR CELLS, from both normal donors and cancer patients, spontaneously lyse a number of cultured cell lines (Takasugi *et al.*, 1973; Herberman & Holden, 1978). This "natural" or spontaneous killing (NK) is, by definition, independent of intentional immunization, although some evidence for target preference, if not clear specificity, at the effector level has been presented (Cannon *et al.*, 1977; Koide & Takasugi, 1977).

There is disagreement in the literature on the precise nature of the effector population(s) in NK, although evidence is accumulating for more than one lymphoid fraction (Kall & Koren, 1978; Potter & Moore, 1979). Thus, whereas earlier studies claimed that NK was mediated either by non-T cells (De Vries *et al.*, 1974; Pross & Jondal, 1975; Jondal & Pross, 1975) or by T cells alone (West *et al.*, 1977; Kay *et al.*, 1977) more recent work has shown that both T and non-T fractions are cytolytic (Gupta *et al.*, 1978). It has also been suggested (West *et al.*, 1977) that spontaneously cytotoxic T cells form rosettes with sheep erythrocytes (SRBC) only under optimal conditions ("low-affinity" T cells). "High-affinity" T-cell fractions prepared under limiting conditions of SRBC concentration and temperature do not lyse even the highly susceptible erythroleukaemia target, K562.

Irrespective of lineage, some controversy persists as to whether all effector populations necessarily express Fc receptors (Kay *et al.*, 1977; Bakacs *et al.*, 1977), complement receptors (Pross & Jondal, 1975; Vessella *et al.*, 1978), both (Jondal & Pross, 1975) or neither (Bolhuis *et al.*, 1978). T cells bearing Fc receptors for IgG, but not for IgM, or Fc-receptor-negative T cells, lyse K562 (West *et al.*, 1977; Gupta *et al.*, 1978). However, although whole mononuclear cells depleted of Fc-receptor-positive cells are similarly not cytotoxic for K562, they do lyse monolayer cell lines, admittedly in a longer-term assay (Bolhuis *et al.*, 1978).

Correspondence to: Dr R. A. Knight, ICRF Human Tumour Immunology Group, University College Hospital Medical School, University Street, WC1E 6JJ.

The effector cells in antibody-dependent cellular cytotoxicity (ADCC) are, again, low-affinity T cells bearing IgG Fc receptors (West *et al.*, 1978; Shaw *et al.*, 1979) and many attempts have been made to correlate or distinguish NK and ADCC effector populations. Indeed, it has been suggested that NK is a particular form of ADCC, mediated by endogenous cytophilic antibody bound to the effector cell through an Fc receptor (Koide & Tagasugi, 1977). NK and ADCC have, however, been differentiated both by selective competition experiments with NK-sensitive or antibody-coated target cells (Koren & Williams, 1978) and in specific immunodeficiency states (Koren *et al.*, 1978). Moreover, Fc-receptor-negative cells can mediate NK but are inactive in ADCC (Bolhuis *et al.*, 1978).

We report here the existence of more than one effector population for a chosen target cell, and show a functional separation of NK effector compartments by target preference. Nylon-non-adherent T cells are cytolytic for 4 commonly used target cell lines, but do not lyse the breast-cancer-derived target, MDA-157. However, other effector fractions, including an adherent T cell, do lyse MDA-157 as well as the other 4 targets. This must imply that NK is an effect contributed by multiple effector components, which are distinguishable by the mechanisms of action.

### MATERIALS AND METHODS

*Peripheral-blood mononuclear cells* (*MNC*).—Blood samples, anticoagulated by preservative-free heparin, were drawn from healthy laboratory staff. The blood was diluted in balanced salt solution (BSS) and centrifuged at 1000 *g* for 15 min over Ficoll–Hypaque at a density of 1·077 g/ml (Böyum, 1968). Mononuclear cells (MNCs) recovered from the diluent–Ficoll interface were washed twice in BSS before further fractionation.

*Preparative E-rosetting.*—1 vol MNCs at $10^7$ cells/ml in BSS were mixed with 0·5 vol heat-inactivated foetal calf serum (FCS; Flow Laboratories, Paisley, Scotland) and with 2 vol of a 2% solution of sheep red blood cells (SRBC; Burroughs Wellcome, Beckenham) which had been treated with neuraminidase (Behringwerke AG, Marburg, West Germany). The mixtures were spun at 130 *g* for 10 min, and incubated at room temperature for 1 h. The pellets were then resuspended by gentle axial rotation, and centrifuged at 650 *g* for 15 min over Ficoll–Hypaque at a density of 1·086 g/ml. E-rosette-negative ($ER^-$) cells, recovered from the interface, and containing less than 1% rosetted cells, were washed twice in BSS. SRBC in the pellet were lysed with distilled water, and the E-rosette-positive ($ER^+$) cells rapidly diluted and washed in BSS.

*Nylon-wool separation.*—Fractionation on nylon-wool columns was performed essentially as described by Julius *et al.* (1973). Six hundred mg nylon wool was packed into 10ml syringe barrels and preincubated for 30 min in RPMI 1640 (ICRF Media Unit, London) containing 10mM glutamine and 10% heat-inactivated FCS (RF10). We had established in preliminary experiments that variation of the applied cell inoculum between $5 \times 10^5$ and $5 \times 10^7$ cells to a column of this capacity made no difference to the proportional recovery of non-adherent (NA) cells, and did not exceed these extremes in the further fractionation of any E-rosetted fraction.

Cells were incubated on nylon-wool columns for 40 min at 37°C. Non-adherent cells were then eluted by running 20 ml RF10 through the column at a speed low enough to prevent the development of a head of medium above the surface of the nylon. The nylon-adherent cells were recovered by 4 cycles of alternately teasing and squeezing the nylon wool in medium.

In the fractionation routinely used the total of E-rosette-forming ($ER^+$) cells were first separated from non-rosette-forming cells ($ER^-$). Both $ER^+$ and $ER^-$ fractions were then separately incubated on nylon-wool columns, and both nylon-non-adherent and nylon-adherent cells recovered.

*Cell cultures.*—MDA-157, a mammary epithelial cell line established from a malignant effusion of a patient with breast cancer (Young *et al.*, 1974) was a kind gift from Dr R. Cailleau (M.D. Anderson Hospital, Texas) to Dr Nancy Hogg.

The Raji cell line—derived from a maxillary Burkitt lymphoma (Pulvertaft, 1965)—and the Chang liver-cell line (Chang, 1954) were both obtained from Flow Laboratories. K562 (Lozzio & Lozzio, 1973), Daudi (Klein

*et al.*, 1968) and Molt 4 (Minowada *et al.*, 1972) were kindly provided by Dr M. Greaves.

MDA-157 and Raji were mycoplasma-free on repeated testing; K562 and Chang cells both contained mycoplasma. All cell lines were maintained in RF10. When confluent, the monolayer cell lines, MDA-157 and Chang, were detached from the flask by a brief treatment with 0·02% versene, and subcultured in RF10. The concentration of suspension cultures was maintained between 1 and $5 \times 10^5$ cells per ml.

Using monoclonal antisera, Molt 4 cells were shown to express HLA-A & B, but not HLA-D; Daudi cells to have HLA-D, but not HLA-A & B; and K562 cells to express minimal amounts of any HLA antigen (kindly assayed by Dr P. C. L. Beverley).

For labelling, target cells were suspended in 200 $\mu$l RF10 at a concentration between 1 and $5 \times 10^7$ cells per ml, and 100 $\mu$Ci sodium [$^{51}$Cr] chromate (CJSI, The Radiochemical Centre, Amersham, England) added. After incubating for 1 h at 37°C, the targets were washed $\times 3$ and finally resuspended at a concentration of $5 \times 10^4$ cells/ml.

*Cytotoxic assay.*—The assay was set up in U-bottomed Linbro microtitre plates (No. 76-311-04; Flow Laboratories, Paisley, Scotland). One hundred $\mu$l of the effector-cell populations in triplicate, generally in 3 doubling dilutions, were mixed with 100$\mu$l volumes of labelled target cells. Release of label from targets incubated in medium alone, and total ct/min incorporated in a 100$\mu$l aliquot of the target-cell suspension were measured in triplicate. The plates were centrifuged, and incubated in an atmosphere of 5% $CO_2$ in air for 14 to 17 h. In some experiments using the K562 target, the incubation was terminated after 4 h.

At the conclusion of the incubation, the plates were centrifuged again. One hundred $\mu$l of supernatant from each well was transferred to individual plastic tubes (LP2; Luckham Ltd, Burgess Hill, Sussex) which were then counted in an LKB-Wallac gamma counter (Model 1280 Ultragamma, LKB, Bromma, Sweden). A computer (Model 1222 Databox) attached to the counter was programmed to calculate percentage cytotoxicity according to the following formula:

$$\% \text{ cytotoxicity} = \frac{\text{ct/min experimental well} - \text{medium ct/min}}{\frac{1}{2} \times \text{total ct/min} - \text{medium ct/min}}$$

as well as the mean ± s.e. of each triplicate group.

Graphs were plotted of percentage cytotoxicity against effector:target-cell ratio. A percentage cytotoxicity value was chosen (generally 20–30%) which lay on the linear portion of the titration of each fraction. Effector:target ratios giving the chosen percentages were read from the graph, and lytic units (LU) per $10^6$ cells calculated according to the formula:

$$\text{LU}/10^6 \text{ cells} = \frac{10^6}{\text{Ratio} \times \text{target cell number } (5 \times 10^3)}$$

*Fraction characterization.*—Surface-membrane $Ig^+$ cells were measured in a direct fluorescence assay, using conjugated goat $F(ab')_2$ anti-human IgG Fab (Nordic Laboratories, Maidenhead, Berkshire). Cells were stained for nonspecific esterase as described by Yam *et al.* (1971).

In vitro *activation.*—$3 \times 10^6$ $ER^+$ non-adherent cells were incubated in 16mm wells in Costar plates (3524 Costar, Cambridge, Mass.) either alone, or with optimal stimulating doses of mitomycin-C-treated Daudi, Molt 4 or K562 cells in a total volume of 2 ml. In previous experiments, the optimal stimulating doses of Daudi, Molt 4 and K562 cells had been established as $10^6$, $10^5$ and $10^6$ cells respectively. For the activation, the RF10 medium was supplemented with $5 \times 10^5$M 2-mercaptoethanol. After incubation for 6 days at 37°C in a 5% $CO_2$, humidified atmosphere, the cultures were harvested, and viable cells recovered by centrifugation over Ficoll-Hypaque. The viable cells were washed twice in BSS, counted and diluted to the required concentration in RF10 for use as effector cells in the cytotoxic assay.

### RESULTS

### *Cell recoveries*

The mean (± s.e.) recovery of peripheral-blood MNCs from 89 Ficoll–Hypaque separations of blood from 23 different normal donors was $1{\cdot}82 \pm 0{\cdot}05 \times 10^6$/ml of whole blood. The yields from some individual donors whose blood has been used on several occasions were significantly different; one donor, used in 21 experiments, gave a mean MNC yield of 2·08 ±

$0 \cdot 10 \times 10^6$/ml; from another, tested on 12 occasions, the mean MNC yield was $1 \cdot 28 \pm 0 \cdot 06 \times 10^6$ cells per ml.

The mean ($\pm$s.e.) combined recovery of $ER^+$ and $ER^-$ cells after preparative rosetting was $86 \cdot 7 \pm 3 \cdot 3\%$ in 46 experiments involving 18 donors. As shown in Table I, a mean of $67 \cdot 4 \pm 1 \cdot 6\%$ of the recovered cells were $ER^+$. Again different individual donors showed a consistent difference in the proportion of $ER^+$ cells. From one donor, tested in 11 experiments, the mean percentage of $ER^+$ cells was $62 \cdot 9 \pm 1 \cdot 7$; the mean proportion of $ER^+$ cells from a second donor was $68 \cdot 6 \pm 3 \cdot 3\%$ in 8 experiments, and from a third, $74 \cdot 4 \pm 3 \cdot 7\%$ in 6 experiments.

TABLE I.—*Fractional cell recoveries after total E-rosetting and nylon-wool adherence**

| Cells applied to E-rosetting | 100† (%) |
|---|---|
| Total cells recovered | 86·7 (100) |
| $ER^+$ | 58·4 (67·4) |
| $ER^-$ | 28·3 (32·6) |
| $ER^+$ applied to nylon | 58·4 (100) |
| Total $ER^+$ recovered | 58·3 (99·8) |
| $Er^+$ non-adherent | 55·3 (94·7) |
| $ER^+$ adherent | 3·0 (5·1) |
| $ER^-$ applied to nylon | 28·3 (100) |
| Total $ER^-$ recovered | 18·8 (66·4) |
| $ER^-$ non-adherent | 7·1 (25·1) |
| $ER^-$ adherent | 11·7 (41·3) |

* Each value is the mean of 22–45 observations.
† The cell numbers recovered ($\times 10^6$) in each fraction are expressed as a proportion of a notional starting mononuclear cell fraction of $100 \times 10^6$ cells.

Table I also shows that all $ER^+$ cells were recovered in the 2 nylon-wool fractions. Only 2/3 of the applied $ER^-$ cells, however, were recovered from the nylon column, possibly due to the strong adherence of some monocytes to the nylon.

### *Cell-fraction characterization*

Surface immunoglobulin fluorescence and nonspecific esterase (NSE) characterization of effector fractions are summarized in Table II. Each value is the mean of 6–10 observations of fractions obtained from 6–9 donors. The $ER^+$ non-adherent fraction is the most homogeneous, containing less than 1% of both B cells and monocytes. This paper principally deals with the spontaneous killing activity of this fraction. The other fractions tested in the NK assay are less pure, the minor $ER^+$ adherent fraction, for example, containing 9% B cells and 4% monocytes. Interestingly, this fraction is depleted, by comparison with the $ER^+$ non-adherent population, for cells with the eccentric-spot NSE staining, characteristic of $T\mu$ cells (Grossi *et al.*, 1978).

TABLE II.—*Surface immunoglobulin (SmIg) and nonspecific esterase (NSE) characterization of cell fractions (as % cells counted)*

| | | NSE | | |
|---|---|---|---|---|
| | SmIg | Spots | Monocytes | Negative |
| Unfractionated | 9·7 | 50·8 | 13·5 | 35·7 |
| $ER^+NA$ | < 1 | 70·3 | 0·3 | 29·4 |
| $ER^+adh$ | 9·2 | 32·4 | 3·8 | 63·8 |
| $ER^-NA$ | 9·8 | 5·0 | 14·8 | 80·2 |
| $ER^-adh$ | 40·4 | 1·2 | 54·3 | 44·5 |

### *Spontaneous cytotoxicity*

The actual level of lysis at a given effector: target ratio on any one target varies in different experiments. However, whereas MDA-157 and Raji are generally similar in their susceptibility to lysis (in 26 experiments, normal MNCs gave a mean of 14·46 LU on MDA-157 and 11·75 on Raji) Chang and K562 are more readily lysed. Since, also, MDA-157 and Raji are mycoplasma-free, we have chosen to compare principally the killing of these 2 targets by the different effector fractions.

Fig. 1 shows titrations of effector populations from the same donor tested on MDA-157 and Raji target cells in the same experiment. We have seen no lysis of either of these targets by the $ER^-$ adherent fraction, and the killing curves of this population are omitted for the sake of clarity. It will be seen that the percentage lysis by the most cytolytic fraction, the $ER^-$ non-adherent, is similar on both targets (35·2% on MDA-157, and 37·2% on Raji at an effector: target ratio of 25/1). The T-enriched, adherent T-cell, and null-cell-enriched fractions all show greater lytic

FIG. 1.—Simultaneous titrations of the cytotoxicity of effector fractions from a single donor on MDA-157 and Raji target cells. Each point is the mean, with s.e. indicated, of triplicate determinations at each effector: target ratio. ■, Unfractionated; ★, $ER^+$, nylon-non-adherent; ▲, $ER^+$, nylon-adherent; ○, $ER^-$, nylon-non-adherent.

activity than unfractionated MNCs on the Raji target. However, the T-enriched $ER^+$ non-adherent fraction does not lyse MDA-157; here, only adherent T cells and the null-cell-enriched fractions are cytotoxic.

In 25 experiments (with 16 different donors) we have seen no convincing cytotoxicity of MDA-157 by the T-enriched fraction. Most of these experiments have compared the lysis by the different effector fractions on MDA-157 and at least one other target. In the majority of these, that other target has been killed by the non-adherent T-cell fraction, whereas in the same experiment, no lysis of MDA-157 by this population has been seen.

On the MDA-157 target, cytotoxicity was enriched in the adherent T-cell fraction, by comparison with the lytic activity of unfractionated MNCs, in 25/26 experiments (96%) and in 18 of these (69%) this was the more enriched fraction. In 89% of experiments the null-cell-enriched fraction also showed greater specific cytolytic activity than unfractionated cells. The null-cell-enriched fraction was the more enriched killer population, however, in only 26% of experiments.

On the Raji target, some fractions, although less cytolytic than the unfractionated MNCs, still show significant and titrable killing. Thus, whereas the T-enriched fraction has killed Raji in 12/14 experiments, in only 8 of these is cytotoxicity greater than in the unfractionated cells. True cytotoxic enrichment was seen in the adherent T-cell and the null-cell-enriched fractions in 5/14 and 11/14 experiments respectively.

Fig. 2 shows the LU on MDA-157 and Raji targets in 2 experiments. In Fig. 2a the data are expressed as $LU/10^6$ cells, giving a measure of the specific cytolytic activity of an effector fraction. In Fig. 2b the total lytic activity for each fraction is shown for the same 2 experiments. Although the specific cytolytic activity of the adherent T-cell fraction on the MDA-157 target is substantial, the total lytic contribution by this fraction is small, since it contains $< 5\%$ of the starting MNCs. On the Raji target, although the $ER^+$ non-adherent ($ER^+$ NA) fraction shows no enrichment of specific activity over the unfractionated MNCs, this fraction, by containing the largest proportion of the starting cells, forms a major component of the total LU of the unfractionated MNCs.

Killing curves on Chang and K562 targets are shown in Fig. 3. On both targets, the null-cell-enriched fraction shows the strongest cytolytic activity, but in both cases the T-enriched fraction shows significant and titrable killing. In one experiment in which the cytotoxicity of the 3 effector fractions from 2 donors was compared on all 4 targets (MDA-157, Raji, Chang, and K562) the enriched T fractions showed significant cytotoxicity on Raji, Chang and K562, but not on MDA-157. On K562, the 3 effector fractions show the same rank order of activity,

FIG. 2.—Lytic units, per $10^6$ cells (a) or as total lytic units per fraction (b), by effector fractions from 2 donors tested in the same assay on MDA-157 and Raji target cells. □ unfractionated MNCs; ⊟ $ER^+$ nylon-non-adherent ($ER^+NA$); ⁝⁝⁝ $ER^+$ nylon-adherent; ▨ $ER^-$ nylon-non-adherent. Since no cytotoxicity by $ER^+NA$ cells was detected on MDA-157, the total lytic units for this fraction can only be expressed as < 125 units, indicated (b) by the symbol [✳].

FIG. 3.—Titrations of the cytotoxicity of effector fractions from a single donor, tested in the same assay, on Chang and K562 target cells. The assay on Chang targets was incubated overnight; on K562, for 4 h. In the same experiment, the $ER^+$, non-adherent fraction did not lyse MDA-157. ■, unfractionated; ●, $ER^+$, non-adherent; ▲, $ER^+$, adherent; □, $ER^-$, non-adherent.

whether the assay is run for 4 h or, using lower effector: target ratios, overnight.

*Cytotoxicity by stimulated $ER^+NA$ cells*

Cytotoxicity by $ER^+NA$ cells, stimulated with Daudi, Molt 4, and K562 cells,

FIG. 4.—Cytotoxicity on MDA-157 target cells of ER+NA cells activated *in vitro* for 6 days with mitomycin-C-treated Daudi cells (■——■), Molt 4 cells (△——△), or K562 cells (▲——▲). Cytotoxicity by unstimulated ER+NA cells cultured for 6 days is also shown (○——○).

on MDA-157 targets is shown in Fig. 4. Unstimulated ER+NA cells are not cytotoxic, whereas cells stimulated with optimal doses of the 3 cell lines show titrating cytotoxicity. In addition, ER+NA cells stimulated by allogeneic ER− cells become cytotoxic for MDA-157. The stimulated, but not unstimulated, ER+NA cells are also cytotoxic on Raji target cells (data not shown). The optimal stimulating cell doses for the induction of ER+NA killing of MDA-157 is the same for the generation of cytotoxicity on Raji targets.

### DISCUSSION

This paper seeks to document 2 observations: firstly, that at least 3 effector populations may be active in human NK; secondly, that these functional subsets can be differentiated by target preference. Two implications follow. More obviously, and confirming results elsewhere in the literature, human NK is a multiple effect, not purely a function of any specialized NK cell. In addition, it follows that if a target like MDA-157 can be lysed by any one of these effector compartments, but not by another, the lytic mechanism may well be different between individual NK populations.

The proportionate yields of total ER+ cells on the preparative scale reported here are rather lower than in some published analytical studies (Hoffman & Kunkel, 1976). However, the significant difference shown in the proportion of total ER+ cells between different donors would suggest that a consistent ER+ fraction is being separated. The T-enriched fraction is almost totally depleted of B cells and monocytes (Table II), and it is with the target selectivity in NK of this population that the paper is principally concerned.

This T-enriched fraction does not lyse MDA-157 in an overnight assay. However, it is cytotoxic for 4 other targets—Raji, Chang, K562 and Molt-4 (data not shown). Two other effector populations, the adherent T cell, and the null-cell enriched, kill all 5 cultured target cell lines.

The levels of killing on MDA-157 and Raji targets are similar, although Chang and K562 (in a 4h assay) are somewhat more readily lysed. This implies that the T-enriched target preference is not a function of differential target lysability. Non-adherent, as well as adherent T cells, kill Chang, which is, like MDA-157, a monolayer target. The absence of T-enriched killing of MDA-157 cannot therefore be explained by the relative lytic efficiency towards suspension as opposed to monolayer targets. Given the equivalent lytic susceptibility of MDA-157 and Raji, the data cannot be explained by the differential representation of a common effector cell in the different fractions but, rather, requires genuine heterogeneity of NK effectors.

T-cell killing of MDA-157 can be generated by culture with stimulating cells expressing either all, part, or none, of the major HLA antigens (Fig. 4). This suggests that the absence of fresh T-cell killing of this target is not due to a clonal deficiency in the ER+NA population.

It has been suggested that NK is a form of ADCC, mediated by cytophilic, presumably "natural" antibody (Koide & Takasugi, 1977). Thus, trypsinization of NK cells, followed by their recovery in serum absorbed with a given target cell, causes the specific loss of cytotoxicity for the absorbing target. Moreover, since there is considerable evidence that at least some NK effector populations express Fc receptors (Hersey *et al.*, 1975; Kay *et al.*, 1977) the binding of antibody by the effector population is readily explicable.

If we assume that the adherent T cell, and the non-T killing of MDA-157 do indeed operate by such a mechanism, we have to explain why the presumed anti-MDA-157 antibody cannot mediate T-enriched killing of this target. Any deficiency can hardly be at the level of antibody binding by the T-enriched effectors, since a postulated cytophilic anti-Raji antibody allows Raji killing by all 3 effector populations described here.

Bolhuis *et al.* (1978) have reached a similar conclusion. Whereas depletion of Fc-receptor-bearing cells completely abrogated both spontaneous killing of K562 and xenogeneic ADCC, little effect was noted on the killing of monolayer carcinoma of the colon and melanoma lines. It would appear, however, that killing through cytophilic antibody attached to Fc-receptor-bearing cells need not be confined to suspension targets since, in terms of the effector populations studied here, no difference in the fractions capable of lysing the monolayer Chang and the suspension Raji and K562 targets was detected.

Many conflicting reports (Herberman & Holden, 1978; Beverley & Knight, 1979) on the nature of the human NK cell(s) have concentrated on the lysis by MNC fractions of a single target. The demonstration here that some effector populations show a clear target preference may help to resolve some of these inconsistencies.

We have shown that whereas 4 of the target cell lines used are lysed by at least 3 NK effector populations, a fifth, MDA-157, is resistant to lysis by one of them. Two of these effector compartments are $ER^+$, distinguished by nylon adherence, and the third, $ER^-$. This dissociation by target preference implies that different NK effectors kill *via* different lytic mechanisms. It further suggests that these mechanistically separate populations can be studied selectively by appropriate choice of target cells.

We would like to thank Dr Peter Beverley and Dr R. L. Souhami for helpful discussions, and all our colleagues for their generous donations of blood. We are also grateful to Virginia Iliescu for expert technical assistance. This work was supported by the Cancer Research Campaign and the Wellcome Trust.

### REFERENCES

Bakacs, T., Gergely, P., Cornain, S. & Klein, E. (1977) Characterisation of human lymphocyte subpopulations for cytotoxicity against tumor-derived monolayer cultures. *Int. J. Cancer*, **19**, 441.

Beverley, P. C. L. & Knight, R. A. (1979) Killing comes naturally. *Nature*, **278**, 119.

Bolhuis, R. L. H., Schuit, H. R. E., Nooyen, A. M. & Ronteltap, C. P. M. (1978) Characterisation of natural killer (NK) cells and killer (K) cells in human blood: discrimination between NK and K cell activities. *Eur. J. Immunol.*, **8**, 731.

Böyum, A. (1968) Separation of leukocytes from blood and bone marrow. *Scand. J. Clin. Lab. Invest.*, **21** (Suppl. 97), 77.

Cannon, G. B., Bonnard, G. D., Djeu, J., West, W. H. & Herberman, R. B. (1977) Relationship of human natural lymphocyte-mediated cytotoxicity to cytotoxicity of breast-cancer-derived target cells. *Int. J. Cancer*, **19**, 487.

Chang, R. S-M. (1954) Continuous subcultivation of epithelial-like cells from normal human tissues. *Proc. Soc. Exp. Biol.*, **87**, 440.

de Vries, J. E., Cornain, S. & Rümke, P. (1974) Cytotoxicity of non-T *versus* T-lymphocytes from melanoma patients and healthy donors on short- and long-term cultured melanoma cells. *Int. J. Cancer*, **14**, 427.

Grossi, C. E., Webb, S. R., Zicca, A. & 4 others (1978) Morphological and histological analyses of two human T-cell subpopulations bearing receptors for IgM or IgG. *J. Exp. Med.*, **147**, 1405.

Gupta, S., Fernandes, G., Nair, M. & Good, R. A. (1978) Spontaneous and antibody-dependent cell-mediated cytotoxicity by human T cell subpopulations. *Proc. Natl Acad. Sci. U.S.A.*, **75**, 5137.

Herberman, R. B. & Holden, H. T. (1978) Natural cell-mediated immunity. *Adv. Cancer Res.*, **27**, 305.

Hersey, P., Edwards, A., Edwards, J., Adams, E., Milton, G. W. & Nelson, D. S. (1975) Specificity of cell-mediated cytotoxicity against human melanoma lines: Evidence for "non-specific" killing by activated T cells. *Int. J. Cancer*, **16**, 173.

Hoffman, T. & Kundel, H. G. (1976) The E-rosette test. In *In Vitro Methods in Cell-mediated and Tumour Immunity*. Eds Bloom & David. New York: Academic Press. p. 71.

JONDAL, M. & PROSS, H. F. (1975) Surface markers on human B- and T-lymphocytes. VI. Cytotoxicity against cell lines as a functional marker for lymphocyte subpopulations. *Int. J. Cancer*, **15**, 596.

JULIUS, M. H., SIMPSON, E. & HERZENBERG, L. A. (1973) A rapid method for the isolation of functional thymus derived murine lymphocytes. *Eur. J. Immunol.*, **3**, 645.

KALL, M. A. & KOREN, H. S. (1978) Heterogeneity of human natural killer cell populations. *Cell. Immunol.*, **40**, 58.

KAY, H. D., BONNARD, G. D., WEST, W. H. & HERBERMAN, R. B. (1977) A functional comparison of human Fc-receptor-bearing lymphocytes active in natural cytotoxicity and antibody-dependent cellular cytotoxicity. *J. Immunol.*, **118**, 2058.

KLEIN, G., PEARSON, G., NADKARNI, J. S. & 5 others (1968) Relation between Epstein–Barr viral and cell membrane immunofluorescence of Burkitt tumour cells. *J. Exp. Med.*, **128**, 1011.

KOIDE, Y. & TAKASUGI, M. (1977) Determination of specificity in natural cell-mediated cytotoxicity by natural antibodies. *J. Natl Cancer Inst.*, **59**, 1099.

KOREN, H. S., AMOS, D. B. & KIM, Y. B. (1978) Natural killing in immunodeficient patients. *J. Immunol.*, **120**, 796.

KOREN, H. S. & WILLIAMS, M. S. (1978) Natural killing and antibody-dependent cellular cytotoxicity are mediated by different mechanisms and by different cells. *J. Immunol.*, **121**, 1956.

LOZZIO, C. B. & LOZZIO, B. B. (1973) Cytotoxicity of a factor isolated from human spleen. *J. Natl Cancer Inst.*, **50**, 535.

MINOWADA, J., OHNUMA, T. & MOORE, G. E. (1972) Rosette-forming human lymphoid cell lines. I. Establishment and evidence for origin of thymus-derived lymphocytes. *J. Natl Cancer Inst.*, **49**, 891.

POTTER, M. R. & MOORE, M. (1979) Natural cytotoxic reactivity of human lymphocyte subpopulations. *Immunology*, **37**, 187.

PROSS, H. F. & JONDAL, M. (1975) Cytotoxic lymphocytes from normal donors: A functional marker of human non-T lymphocytes. *Clin. Exp. Immunol.*, **21**, 226.

PULVERTAFT, R. J. V. (1965) A study of malignant tumours in Nigeria by short-term culture. *J. Clin. Pathol.*, **18**, 261.

SHAW, S., PICHLER, W. J. & NELSON, D. L. (1979) Fc receptors on human T-lymphocytes. *J. Immunol.*, **122**, 599.

TAKASUGI, M., MICKEY, M. R. & TERASAKI, P. I. (1973) Reactivity of lymphocytes from normal persons on cultured tumour cells. *Cancer Res.*, **33**, 2898.

VESSELLA, R. L., GORMUS, B. J., LANGE, P. H. & KAPLAN, M. E. (1978) Heterogeneity among human lymphocyte effector cells mediating spontaneous lymphocyte-mediated cytotoxicity. *Int. J. Cancer*, **21**, 594.

WEST, W. H., BOOZER, R. B. & HERBERMAN, R. B. (1978) Low affinity E-rosette formation by the human K cell. *J. Immunol.*, **120**, 90.

WEST, W. H., CANNON, G. B., KAY, H. D., BONNARD, G. D. & HERBERMAN, R. B. (1977) Natural cytotoxic reactivity of human lymphocytes against a myeloid cell line: Characterisation of effector cells. *J. Immunol.*, **118**, 355.

YAM, L. T., LI, C. Y., CROSBY, W. H. (1971) Cytochemical identification of monocytes and granulocytes. *Am. J. Clin. Pathol.*, **55**, 283.

YOUNG, R. K., CAILLEAU, R. M., MACKAY, B. & REEVES, W. J. (1974) Establishment of epithelial cell line MDA-MB-157 from metastatic pleural effusion of human breast carcinoma. *In vitro*, **9** 239.